# Interpretation of Ligand-Based Activity Cliff Prediction Models Using the Matched Molecular Pair Kernel

**DOI:** 10.3390/molecules26164916

**Published:** 2021-08-13

**Authors:** Shunsuke Tamura, Swarit Jasial, Tomoyuki Miyao, Kimito Funatsu

**Affiliations:** 1 Graduate School of Science and Technology, Nara Institute of Science and Technology, 8916-5 Takayama-cho, Ikoma 630-0192, Japan; tamura.shunsuke.tn0@ms.naist.jp (S.T.); jasial@dsc.naist.jp (S.J.); miyao@dsc.naist.jp (T.M.); 2 Data Science Center, Nara Institute of Science and Technology, 8916-5 Takayama-cho, Ikoma 630-0192, Japan

**Keywords:** chemoinformatics, activity-cliff, support vector machine, model interpretation, SHapley Additive exPlanations, matched molecular pair

## Abstract

Activity cliffs (ACs) are formed by two structurally similar compounds with a large difference in potency. Accurate AC prediction is expected to help researchers’ decisions in the early stages of drug discovery. Previously, predictive models based on matched molecular pair (MMP) cliffs have been proposed. However, the proposed methods face a challenge of interpretability due to the black-box character of the predictive models. In this study, we developed interpretable MMP fingerprints and modified a model-specific interpretation approach for models based on a support vector machine (SVM) and MMP kernel. We compared important features highlighted by this SVM-based interpretation approach and the SHapley Additive exPlanations (SHAP) as a major model-independent approach. The model-specific approach could capture the difference between AC and non-AC, while SHAP assigned high weights to the features not present in the test instances. For specific MMPs, the feature weights mapped by the SVM-based interpretation method were in agreement with the previously confirmed binding knowledge from X-ray co-crystal structures, indicating that this method is able to interpret the AC prediction model in a chemically intuitive manner.

## 1. Introduction

Activity cliffs (ACs) [1] are formed by two structurally similar compounds with a large difference in potency. ACs can be found in hit-to-lead or lead optimization phases in which structurally analogous compounds are examined to obtain compounds with the desired potency or properties such as absorption, distribution, metabolism, excretion, and toxicity. The existence of ACs indicates the discontinuity of the structure–activity relationship and prevents efficient lead optimization. Conversely, AC would have substantial information on the protein–ligand interaction and therefore ACs are of high interest in medicinal chemistry.

A useful tool to represent the similarity between compounds with small chemical modifications is the matched molecular pair (MMP) [2]. An MMP is composed of two structurally similar compounds that share a common substructure (core) and differ at a single site (substituents). MMP is helpful to link the potency change and single chemical modification, and to systematically identify ACs as MMP-cliffs [3]. An MMP-cliff is defined as an MMP with a significant difference in potency (generally >2 log units).

In recent years, ligand-based AC prediction, which aims to predict the formation of AC between two ligand compounds with only their chemical property or structural features, has been extensively explored [4,5,6,7,8]. Machine learning approaches have attempted to predict ACs. However, ACs are fundamentally difficult to predict by machine learning due to their discontinuity; a small difference in input makes a large difference in output [4]. That is why traditional quantitative structure–activity relationship (QSAR) models, which construct statistical models between compounds and their potency, have failed to predict them accurately [9]. To overcome this limitation, models were constructed based on the structural features of paired analogous compounds to predict their potency difference and information of not only the core but also substituent transformation was utilized [6,7]. In this approach, the AC prediction model was constructed with the help of a support vector machine [10], fingerprints representing the features in core and substituents independently (MMP fingerprints), and a well-defined kernel function (MMP kernel) for evaluating the similarity between MMPs. An MMP kernel is a product of the Tanimoto kernel [11] for the core and substituents. As a result of this study, for specific targets, ACs having analogous core structures were predictable by using a specific molecular representation and similarity evaluation approach [7]. In our previous study, we further improved the performance of AC prediction by defining the applicability domain based on the similarity of fingerprints, considering the environment around the attachment point of the core and substituents [8,12]. However, due to the black-box character of the SVM, the predictive models were not interpretable.

Though interpretability of machine learning models is of high importance in any scientific field, interpretable AC prediction models have not been explored much. Lindberg and Lee proposed a model-independent approach termed as the SHapley Additive exPlanations (SHAP) to interpret the contribution of each feature to an output [13]. SHAP can be regarded as an extension of local interpretable model-agnostic explanations (LIME) [14]. SHAP approximates each output of black-box models, such as the SVM with a linear local explanation model in which weights are Shapley values in game theory. SHAP is being used in this field due to its robust mathematical background and easy-to-apply library developed by the authors [15,16,17,18]. There is a model-specific approach proposed by Balfer et al. to interpret an output from the SVM with the Tanimoto kernel [19]. This method obtains feature contributions by transforming the kernel function into the sum of feature weights similar to the linear kernel. However, this method cannot be applied to the predictive AC model with the SVM because the kernel function used for similarity evaluation between MMPs cannot be transformed into the sum of feature weights.

In this study, for interpreting AC prediction models, we developed the MMP fingerprint-based model-specific approach. The main idea is to decompose the cross-term contribution of the MMP kernel into core and substituents evenly, which enables us to approximate this kernel as the sum of feature weights. This interpretation method was applied to well-performed AC prediction models using the SVM and MMP kernel for thrombin and carbonic anhydrase II. SHAP was also applied to the same models as a control calculation. In qualitative analyses, the features with high contribution indicated by the SVM-based approach were reasonable, while those by SHAP were difficult to interpret because SHAP gave high weights to the features not inside the target molecule. The X-ray co-crystal structure for a particular target was analyzed in order to validate features highlighted by the SVM-based interpretation approach. This study sheds light on the limitation of SHAP and enables us to interpret the AC prediction model in a chemically intuitive manner.

## 2. Result and Discussion

### 2.1. Performance of the AC Prediction Models

The AC prediction models were constructed for the thrombin (thr) and carbonic anhydrase II (ca2), which showed high performances in our previous study. A matched molecular series (MMS) was iteratively selected as the test set and the rest of the MMSs were used as the training set. This procedure was performed until every MMS was selected as the test set. MMPs consisting of compounds shared with the training set were discarded to ensure that the test set did not share the same core and to fairly evaluate the extrapolation ability. Overall, the performance of the AC prediction models for the targets were high. The values of recall, the area under the curve of the receiver–operator characteristic (AUC ROC), and Matthew’s correlation coefficient (MCC) were 0.98, 0.69, and 0.89 for thr, and 0.86, 0.46, and 0.69 for ca2, respectively. The constructed AC prediction models were predictive enough, which was consistent with the previous study, though the MMP fingerprint generation procedure was slightly modified for interpretability.

### 2.2. Interpretability of the Model

**Figure 1:**
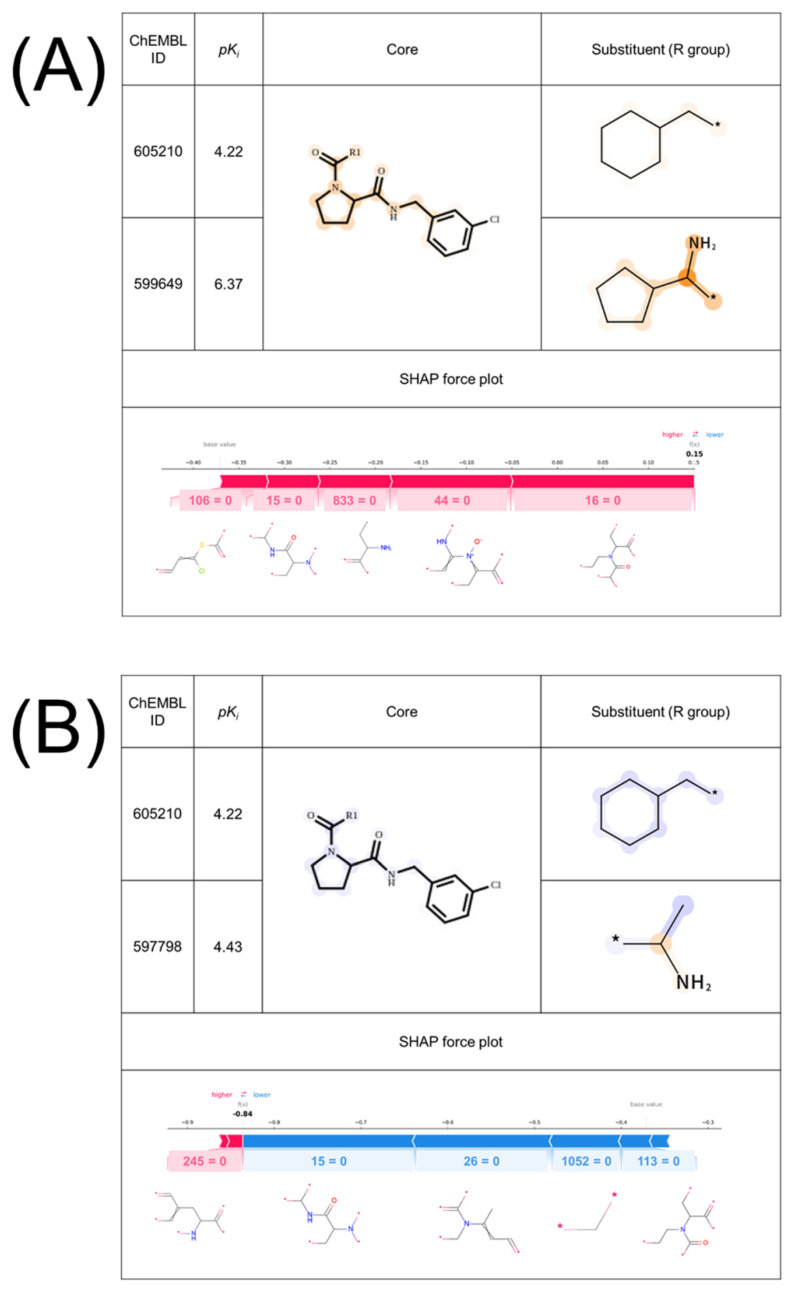
**Comparison of the feature contribution mapping and SHAP for exemplary accurately predicted MMPs of thrombin.** Exemplary (**A**) TP and (**B**) TN MMPs of thrombin with the feature contribution map and SHAP force plot for the MMPs are shown. MMPs are formed by (**A**) ChEMBL605210 and ChEMBL599649, and (**B**) ChEMBL605210 and ChEMBL597798. The core and substituent of the low potency compound in these MMPs were fixed to validate whether the model could capture the difference concerning the high potency compound. The orange color represents positive contribution and the blue color represents negative contribution. For the feature contribution mapping, higher contribution towards the predicted results is represented by a darker color. In the SHAP force plots, the baseline and output value (bold) are shown. The presence or absence of the substructures corresponding to the feature numbers are also shown as one or zero, respectively. The substructures corresponding to the feature numbers are shown below the plot.

Figure 1 shows two exemplary MMPs for thr predicted as true positive (TP) (Figure 1A) and true negative (TN) (Figure 1B) with the feature contributions mapped onto the structures and the corresponding SHAP force plots. For the SVM-based interpretation approach, the higher the positive or negative feature contribution of an atom or a group, the darker the orange or blue color was represented on their structures, respectively. For SHAP, the color in the force plot depicts whether assigned Shapley values are positive (red) or negative (blue) and the length of the bars corresponds to how each feature contributed to the prediction. The MMPs depicted in Figure 1A, B share the low potency compound (ChEMBL605210). The only difference between these MMPs is the substituents of the high potency compounds; the cyclopentenyl group (A) and the methyl group (B). This structural difference would be responsible for the formation of AC. For the SVM-based interpretation approach, positive contributions were assigned to the part of the cyclopentenyl group and negatives to the methyl group. Conversely, SHAP assigned Shapley values to the features missing in both MMPs. From this result, the models have learned the features that would be related to the formation of ACs and the SVM-based interpretation approach could interpret the model that SHAP could not.

Figure S1 shows three other exemplary MMPs of thr predicted as TP (Figure S1A), TN (Figure S14B), and false negative (FN) (Figure S1C). In a similar manner as in Figure 1, the MMPs share the core and one substituent (center) of the low potency compound (ChEMBL598415). According to the force plot, the Shapley values were assigned to the features not present in the MMP as in Figure 1. For the MMP in Figure S1C, the model incorrectly predicted it as non-AC. The SVM-based interpretation approach seemed rational due to the consistent feature contribution for the common features among the MMPs, unlike SHAP. Furthermore, the SVM-based approach could derive a possible explanation for the incorrect prediction for the MMP in Figure S1C. According to the mapped contribution, the contribution of the 1,1-dimethyl ethyl group, which is the different part compared to the other MMPs, is underestimated; therefore, it can be considered that the model has not learned how this substituent affects the formation of ACs.

The same situation was observed in the prediction for ca2. Figure S2 shows three exemplary MMPs of ca2 predicted as TP (Figure S2A), TN (Figure S2B), and false positive (FP) (Figure S2C). In the same manner as above, SHAP did not assign contributions to the features present in the MMPs and did not capture the substructure transformation. For the MMP in Figure S2C, very few training AC MMPs having a highly similar core and similar substructure transformation of the MMP (C) were found. This might be the reason for the misclassification.

### 2.3. Validation of Important Features with X-ray Co-Crystal

**Figure 2:**
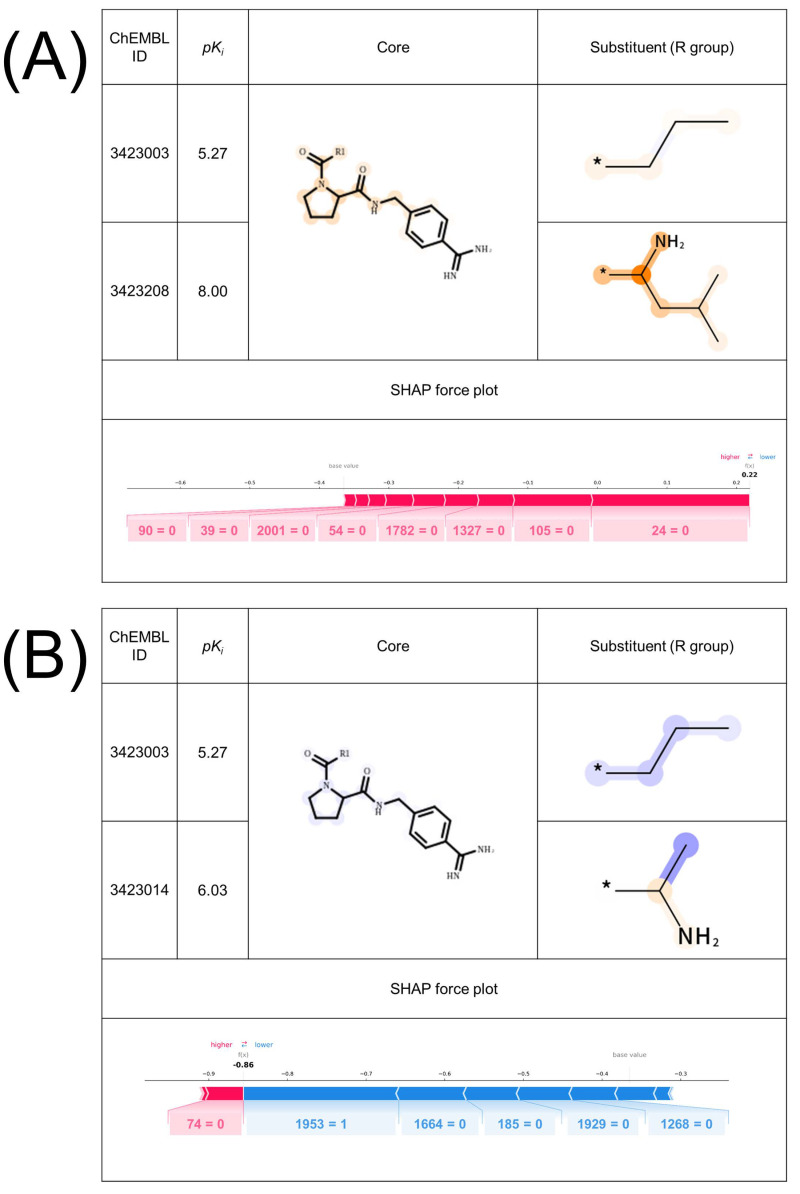
**Comparison of the feature contribution mapping and SHAP for exemplary thrombin MMPs with an X-ray co-crystal structure predicted as TP and TN**. Exemplary (**A**) TP and (**B**) TN MMPs of thrombin with the feature contribution map and SHAP force plot for the MMPs are shown. MMP (**A**) has an X-ray co-crystal structure. MMPs are formed by (**A**) ChEMBL3423003 and ChEMBL3423208, and (**B**) ChEMBL3423003 and ChEMBL3423014.

**Figure 3:**
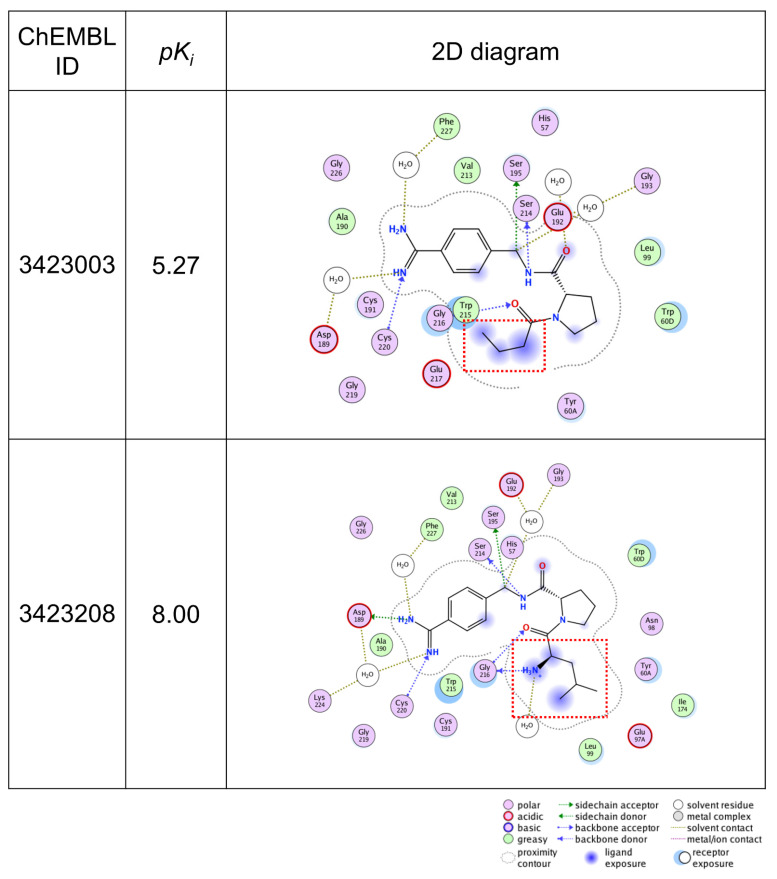
**2D diagrams of the thrombin–ligand interaction based on X-ray co-crystal analysis.** 2D diagrams of the interaction of thrombin and the compounds that were used in the MMP in Figure 2A are illustrated. The ligand compounds are the low potency compound (**top**) and the high potency compound (**bottom**). The structural difference between the two compounds is emphasized by the red squares. The interaction pose was extracted from PDB and the diagram was generated by MOE.

Figure 2 shows two exemplary MMPs for thr predicted as TP (Figure 2A) and TN (Figure 2B). The difference between the MMPs is the 2-methyl propyl group (Figure 2A) and the methyl group (Figure 2B). This substructure transformation is highlighted using the SVM-based interpretation method, while SHAP did not capture the difference. To further investigate whether the highlighted features are rational or chemically intuitive, an MMP (Figure 2A), which forms AC, was compared to the X-ray co-crystal structures retrieved from PDB (PDBIDs:2ZI2, 2ZNK), identified in a previous study [20]. Figure 3 illustrates the 2D map of the interaction between thr and the compounds forming the MMP in Figure 2A. ChEMBL 3423003 (top) has a lower potency and ChEMBL 3423208 (bottom) has a higher potency. From the 2D map, two major aspects that improved the potency are observed. First, when an amine group was substituted on the carbon atom next to the attachment point, a hydrogen bond was formed between the glycine 236 and that amine. Second, when a 1,1-dimethyl ethyl group was substituted on the same carbon atom, the ligand exposure decreased. These two factors are positively highlighted in Figure 2A. The authors reported that the complete dual ladder β-sheet-like hydrogen bond network between the ligand and Gly216 was formed by introducing an amide branch and substituting a 1,1-dimethyl ethyl group instead of a short alkyl chain, contributing to the release of a water molecule in the active pocket, which supports our analysis [21]. This insight also supports the negative feature weight assigned to the methyl group on ChEMBL3423104 in Figure 2B. Thus, the AC prediction models using the proposed MMP fingerprints have the potential to learn the rationale and important features related to AC formation, focusing on the existing features inside the molecules. The SVM-based interpretation method enabled us to see whether the output was reliable and could be useful for checking whether the models have learned chemical knowledge or not.

### 2.4. Limitations of SHAP for AC Prediction Model Interpretability

So far, the interpretability of the SVM-based method and SHAP is discussed by comparing the important features identified by the two methods. SHAP assigned Shapley values to the features missing in the test MMPs. Originally, Shapley values indicate how much the features contribute to the difference between the actual prediction and the expected values of the prediction. In the context of this study, Shapley values quantified the impact of the presence or absence of a substructure on the difference between the actual output of the decision function and the mean output. Therefore, it is natural that the absence of a specific feature improves the possibility of AC formation. Moreover, during the calculation process of SHAP, it is considered that sampling the random value to evaluate the prediction without some features makes SHAP uninterpretable in this study. Fingerprints having features replaced with random values cannot be assembled to actual chemical structures, and the predicted values using the fingerprints are unreliable. However, sampling random values is necessary because unbiased sampling is the key for deriving Shapley values. Further discussion of this fact has not been reported yet. Conversely, the SVM-based interpretation method calculates feature contribution for the features only present in the test MMPs. This is in accordance with chemists’ ways of interpreting inter-molecular interactions, i.e., only existing protein–ligand interactions are counted. Small molecules generally bind with targeted protein residues at a specific substructure or at atoms of the molecule. Model interpretation highlighting this phenomenon can be easily accepted. Even if a machine learning model actually predicts ACs based on the substructures not present inside the molecules, it is hard to utilize this information for further chemical optimization. However, when predicting negative compounds (non-ACs), explanations from both directions seem acceptable because the features missing in the non-ACs might be crucial for producing strong interactions against the target protein. In addition, interpretation of the models for non-toxic compounds and non-active compounds might be in the same category.

For the purpose of interpreting AC prediction models, in particular for AC MMPs, the SVM-based method was informative and could be helpful in scientists’ decision-making.

## 3. Materials and Methods

### 3.1. Data Sets

Two protein targets (thrombin and carbonic anhydrase II) were selected for our analysis as AC prediction models for these targets showed high performance in our previous research [8]. Compounds active against the targets were retrieved from ChEMBL (version 24, European Bioinformatics Institute of the European Molecular Biology Laboratory, Hinxton, UK) [22] and curated with the same procedure as in the previous study [8]. MMP-cliffs were used as the AC definition. MMPs with differences in potency larger than two log units were labeled as AC and less than one as non-AC. The rest of the MMPs were discarded to avoid the inclusion of ambiguous data. MMPs for each target were computationally generated using Hussain and Lee’s approach [23] implemented by Wawer and Bajorath [24]. For MMP generation, substructure exchange was restricted to no more than 13 heavy atoms and the maximum difference between substituents of compounds was restricted to no more than 8 heavy atoms. MMPs having the same core were grouped as the matched molecular series [25]. MMSs composed of only AC MMPs or non-AC MMPs were discarded. The profile of data sets for the selected targets is shown in Table 1.

### 3.2. MMP Fingerprints

**Figure 4:**
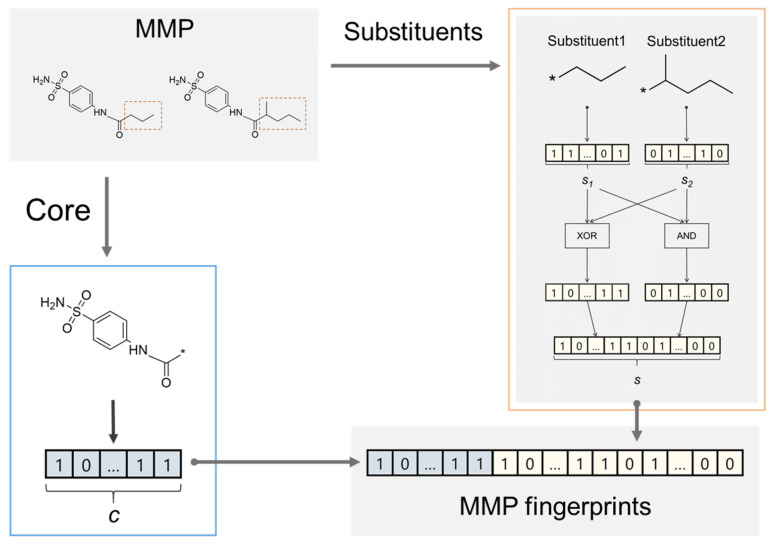
**Fingerprint generation for MMPs.** An MMP is formed by two compounds that share a common scaffold (core) and individual substituents. From each MMP, one core and a set of two substituents were extracted, and then fingerprints were generated for each part. Two types of fingerprints were concatenated to obtain fingerprints representing substituents. One is calculated by taking the XOR operation of two fingerprints of given substituents and the other is calculated by taking the AND operation to represent specific and common features. Finally, MMP fingerprints were generated by concatenating fingerprints for the core and substituents.

MMP fingerprints were generated from MMPs according to the procedure depicted in Figure 4. Fingerprints representing the core and substituent parts were individually calculated for each MMP. Extended connectivity fingerprints of bond diameter 4 (ECFP4) [26] were used as the molecular representation. Features within bond diameter 1 were not used in ECFP4 calculation to clarify the contributions of features over bond diameter 2. For each part, all identifiers corresponding to the features were sorted in ascending order and assigned to bits in the fingerprint vectors in the same order; therefore, the length of MMP fingerprints depended on the target. This was done to prevent feature collision, to ensure the availability of the features for predicting AC, and to calculate feature contribution. The substituent fingerprints were composed of fingerprints calculated by taking the XOR operation and AND operation for each substituent feature vector to represent unique and common features in the two substituents separately and focus on the transformation.

### 3.3. Construction and Evaluation of the AC Prediction Model

An AC prediction model was constructed with the help of a support vector machine. The SVM is a supervised learning approach that distinguishes two classes using a hyperplane obtained by maximizing the margin between the hyperplane and support vectors. This method was originally developed for linear classification problems but the kernel function enables the use of this method for non-linear classification problems. In the present study, the MMP kernel, which is the product of the Tanimoto similarity of the cores and the substituents, was used as a kernel function.

AC prediction was conducted by MMSs. One MMS was selected as a test set and the rest of the MMSs were used as training set. For each test set, MMPs that were composed of compounds used for forming the MMP in the training set were eliminated. After a prediction for the selected test MMS was completed, other MMS were selected as the test set and predicted for. This iterative prediction was performed until all MMSs were selected as the test set. This operation was done to evaluate the extrapolation ability by preventing the sharing of the same core between the test and training sets. In our previous study, two models were constructed with different MMP fingerprint orders for substituents. The predicted output was defined based on both outputs of the models to identify MMPs whose potency improved due to the transformation. However, in this study, because we focused on the model interpretation, only one type of fingerprint order was used for further discussion. The hyperparameter C in each SVM model was optimized using five-fold cross-validation. The performances of the AC prediction models were evaluated using recall, AUC ROC, and MCC.

### 3.4. Feature Contributions for the Tanimoto Kernel in SVM

Our proposed method for feature contribution is based on the feature weighting method for the Tanimoto kernel-based SVM proposed by Balfer and Bajorath [19].

When the SVM is used for non-linear classification tasks, the test instances *x_ts_* are classified based on the following decision function with the kernel function:

(1) $$f\left(x\right)=sign\left(\overset{n}{\underset{i=1}{\sum }}\alpha^{\left(i\right)}y^{\left(i\right)}K\left(x_{tr}^{\left(i\right)},x\right)-b\right)\;$$

where $\;\alpha^{\left(i\right)}$ are Lagrangian multipliers on the dual problem of the objective function to identify the optimal hyperplane; *x_tr_* is the training set composed of *n* samples with the corresponding binary class label *y*; and *b* is the bias term of the calculated hyperplane.

When the linear kernel is used as a kernel function, the feature contribution is easily calculated because Equation (1) can be represented as the sum of feature contributions weighted by $\alpha^{\left(i\right)}y^{\left(i\right)}$ and bias b:

(2) $$f\left(x\right)=sign\left(\underset{d\;\in \;features}{\sum }fc_{linear}\left(x,d\right)-b\right)\;$$

(3) $$fc_{linear}\left(x,d\right)=\overset{n}{\underset{i=1}{\sum }}\alpha^{\left(i\right)}y^{\left(i\right)}x_{tr\;d}^{\left(i\right)}x_{d}\;$$

To facilitate further explanation, we introduce the fragment feature contribution (*frag_fc*), which is one term of feature contribution. *frag_fc* and $fc_{linear}$ can be represented as follows:

(4) $$frag\_fc_{linear}\left(x,d,i\right)=\alpha^{\left(i\right)}y^{\left(i\right)}x_{tr\;d}^{\left(i\right)}x_{d}$$

(5) $$fc_{linear}\left(x,d\right)=\overset{n}{\underset{i=1}{\sum }}frag\_fc_{linear}\left(x,d,i\right)$$

When a non-linear kernel function is used, the decision function is not represented as the sum of feature contribution and bias. The Tanimoto kernel is a good example and is one of the widely used non-linear kernels for molecular property prediction using fingerprints. The Tanimoto kernel is defined as:

(6) $$K_{Tanimoto}\left(u,v\right)=\frac{\langle u,v\rangle }{\langle u,u\rangle +\langle v,v\rangle -\langle u,v\rangle }\;=\overset{D}{\underset{d=1}{\sum }}\frac{u_{d}v_{d}}{\langle u,u\rangle +\langle v,v\rangle -\langle u,v\rangle }$$

The decision function with the Tanimoto kernel can be represented as the sum of feature contributions and the bias as the denominator of the kernel function is constant for individual kernel calculations. Considering this condition, the fragment feature contribution and feature contribution is represented as follows based on the linear kernel:

(7) $$frag\_fc_{Tanimoto}\left(x,d,i\right)=\frac{\alpha^{\left(i\right)}y^{\left(i\right)}x_{tr\;d}^{\left(i\right)}x_{d}}{\langle x_{tr}^{\left(i\right)},x_{tr}^{\left(i\right)}\rangle +\langle x,x\rangle -\langle x_{tr}^{\left(i\right)},x\rangle }$$

(8) $$fc_{Tanimoto}\left(x,d\right)=\overset{n}{\underset{i=1}{\sum }}frag\_fc_{Tanimoto}\left(x,d,i\right)\;$$

To introduce fragment feature contribution, the decision function using the Tanimoto kernel is calculated as the sum of all fragments of feature contribution, defined as:

(9) $$f\left(x\right)=sign\left(\underset{d\;\in \;features}{\sum }fc_{Tanimoto}\left(x,d\right)-b\right)$$

### 3.5. Feature Contributions for the MMP Kernel

SVM using the MMP kernel has been used for AC predictions due to its high performance. The MMP kernel, however, cannot be represented as the sum of feature contributions because it is the product of core-wise and substituent-wise Tanimoto kernel functions, defined as:

(10) $$\begin{array}{l} K_{MMP}\left(u,v\right)\\ =K_{Tanimoto}\left(u_{c},v_{c}\right)\times K_{Tanimoto}\left(u_{s},v_{s}\right)\\ =\frac{\langle u_{c},v_{c}\rangle }{\langle u_{c},u_{c}\rangle +\langle v_{c},v_{c}\rangle -\langle u_{c},v_{c}\rangle }\times \frac{\langle u_{s},v_{s}\rangle }{\langle u_{s},u_{s}\rangle +\langle v_{s},v_{s}\rangle -\langle u_{s},v_{s}\rangle }\\ =\overset{D_{c}}{\underset{d_{c}=1}{\sum }}\frac{u_{c\;d_{c}}v_{c\;d_{c}}}{\langle u_{c},u_{c}\rangle +\langle v_{c},v_{c}\rangle -\langle u_{c},v_{c}\rangle }\times \overset{D_{s}}{\underset{d_{s}=1}{\sum }}\frac{u_{s\;d_{s}}v_{s\;d_{s}}}{\langle u_{s},u_{s}\rangle +\langle v_{s},v_{s}\rangle -\langle u_{s},v_{s}\rangle }\; \end{array}$$

where the subscripts of *c* and *s* represent the core and substituent parts of MMP fingerprints, respectively. For the MMP kernel, two parts of the feature contributions can be individually represented as Equation (7) and a cross-term of features appears by the product of two feature contributions from the two kernels, represented as:

(11) $$\begin{array}{l} frag\_fc_{MMP}\left(x,d_{c},d_{s},i\right)\\ \;\;\;\;\;=\frac{\alpha^{\left(i\right)}y^{\left(i\right)}x_{tr,c\;d_{c}}^{\left(i\right)}x_{c\;d_{c}}}{\langle x_{tr,\;c}^{\left(i\right)},x_{tr,c}^{\left(i\right)}\rangle +\langle x_{c},x_{c}\rangle -\langle x_{tr,c}^{\left(i\right)},x_{c}\rangle }\\ \;\;\;\;\;\times \frac{x_{tr,s\;d_{s}}^{\left(i\right)}x_{s\;d_{s}}}{\langle x_{tr,\;s}^{\left(i\right)},x_{tr,s}^{\left(i\right)}\rangle +\langle x_{s},x_{s}\rangle -\langle x_{tr,s}^{\left(i\right)},x_{s}\rangle }\; \end{array}$$

**Figure 5:**
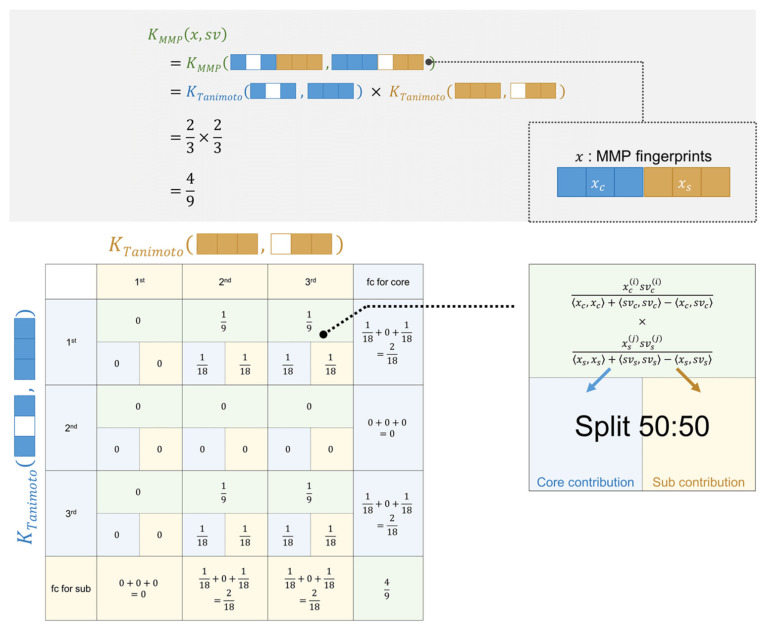
**Assignment of feature contribution based on the MMP kernel.** Shown above is an example of MMP kernel calculation and the assignment of feature contribution from a test MMP (*x*) and a support vector (*sv*) that has three features for the core and three features for substituents. Colored and uncolored squares represent features that are present and absent in MMPs, respectively. The MMP kernel is a product of the Tanimoto kernels of the fingerprints of the core (blue) and substituent (orange) parts that are divided from MMP fingerprints. Half of the cross-term feature weightings are assigned to the features in the core and half to the substituent features.

In order to represent an MMP kernel value as the sum of feature contributions, we propose to decompose the cross-term into half and assign them to a core feature and a substituent feature equally so that the sum of the linear contributions of all the features equals to the SVM output. Figure 5 shows an exemplary case with one support vector (*sv*) and one test instance (*ts*). Each vector has three features for the core and another three for the substituents. The test instance has the first and third feature present in the core and all three features in the substituents. The support vector has all the features present in the core and the second and third feature in the substituents. The matrix in Figure 5 shows how each *frag_fc_Tanimoto_* contributes to the whole MMP kernel. The row corresponds to *frag_fc_Tanimoto_* of the core and the column corresponds to *frag_fc_Tanimoto_* of the substituents. Considering the value of the MMP kernel is a sum of the cross-terms of *frag_fc_Tanimoto_* of the core and substituents, the contribution of a single feature cannot be calculated. To represent the MMP kernel as the sum of the fragments of feature contributions, each cross-term, namely *frag_fc_MMP_*, is divided into the corresponding features for the core and substituents. For instance, at the upper right corner, the cross-term is one-ninth calculated from *frag_fc_Tanimoto_* for the first feature in the core and that for the third feature in the substituents, and half of the cross-term value is assigned to the first feature of the core and another half is assigned to the third feature of the substituents. After dividing the cross-terms, the feature contribution for a feature can be calculated by summing up the divided values along the axis. Using this operation, the MMP kernel is represented as the sum of feature contributions and can be interpreted in the same way as using the simple Tanimoto kernel. Feature contributions were mapped onto the predicted MMP with the same method as in the previous study [19]. The calculated feature contribution for a substructure was distributed for its corresponding atom and bond evenly. Each of the atoms and bonds appeared in substructures more than once; therefore, the weight for an atom or bond was calculated by accumulating the contribution deriving from different substructures. The weights for atoms and bonds are color-coded: red represents positive, blue represents negative, and white represents a weight of zero. The darkness of color-coding corresponds to the magnitude of the assigned weight.

### 3.6. SHAP Theory

SHAP aims to explain the output of a model by computing Shapley values for each feature as the feature contribution based on the coalitional game theory. The Shapley value was originally proposed to fairly distribute the payout among the players along with their contribution to the games. In the context of this study, the players correspond to the features in fingerprints and the payouts correspond to the output of the decision function. In the SHAP algorithm, the output for a test instance is approximated with a local explanation model, defined as:

$$g\left(x^{\prime }\right)=\phi_{0}+\overset{N}{\underset{i=1}{\sum }}\phi_{i}x_{i}^{’}$$

where *g* is the local explanation model; $x^{\prime }\in {\left\{0,1\right\}}^{N}$ takes one when the feature is present in the test instance and zero when absent; *N* is the number of features; and $\phi_{i}\in R$ is the Shapley value as a contribution for feature *i*. All possible combinations of the feature set without the *i*th feature are needed to calculate exact Shapley values for the features and this calculation is highly expensive due to the enormous feature subset. Thus, kernel SHAP is used to efficiently estimate the Shapley values. Kernel SHAP needs a dataset to construct a linear regression model whose coefficients equals Shapley values. First, $x^{\prime }$ representing feature presence or absence is generated *K* times and then $x^{\prime }$ is transformed into feature vectors using mapping function *h* which maps to the corresponding feature in the test instance if a bit is set on (present) or to a random value sampled from the marginal distribution if set off (absent). Subsequently, the linear regression model is constructed as local explanation model *g* so that $g\left(x^{\prime }\right)=f\left(h\left(x^{\prime }\right)\right)$, where *f* is the original model. The model *g* is optimized by minimizing the squared loss weighted by the SHAP kernel. The coefficients of the model *g* are the Shapley values defined as the feature contributions. $\phi_{0}$ is the expected explanatory value (baseline) that represents an expected output value if all input features do not exist. This baseline is calculated as the mean of the objective variable over the training instances.

## 4. Conclusions

MMP-cliff based AC prediction has been explored and predictive models have been proposed previously. However, these models experience a lack of interpretability because of the black-box character. In this study, we developed interpretable MMP fingerprints and modified a model-specific approach to interpret a well-constructed AC prediction model using the SVM and MMP kernel. The important features highlighted by this interpretation method and SHAP were compared to each other. Almost all of the high Shapley values were assigned to features not present in the test instance, which indicates that SHAP is not helpful to obtain a chemically intuitive interpretation. Conversely, the SVM-based method highlighted features present in the test instance. To validate the features highlighted by this method, an X-ray co-crystal structures for a particular target were analyzed and it accurately assigned a high contribution to the features related to hydrogen bonding and ligand exposure. This study sheds light on the limitation of SHAP and enables us to interpret the AC prediction model in a chemically intuitive manner using a model-specific approach.

## Figures and Tables

**Table 1:** Data sets.

ChEMBL ID	Target	Abbreviation	#CPDs	#MMPs	#AC	#MMSs	Potency (pK_i_)		MW		#HA	
							Max	Min	Max	Min	Max	Min
204	Thrombin	thr	221	839	311	29	10.30	2.81	693.86	280.75	50	19
205	Carbonic anhydrase II	ca2	362	989	248	70	11.00	0.70	678.36	153.14	42	11

For each data set, the CHEMBL ID, number of compounds (#CPDs), number of MMSs (#MMSs), maximum and minimum potencies (pK_i_ values), maximum and minimum molecular weight (MW), and heavy atom counts (#HA) are reported.

## Data Availability

The data sets in this study are available in the Supplementary Materials.

## Supplementary Materials

The following are available online, Figure S1: Comparison of feature contribution mapping and SHAP for exemplary thrombin MMPs predicted as TP, TN, FN. Figure S2: Comparison of feature contribution mapping and SHAP for exemplary ca2 MMPs predicted as TP, TN, FP. Datasets.xlsx: two sheets corresponding to the target proteins. Each row has the SMILES for a core, substituent1 and substituent2, potency difference between the compounds of an MMP, the class label of AC (represented as 1) or non-AC (represented as −1), and the MMS ID.
