# Supplementary material for: Interpretation of Ligand-Based Activity Cliff Prediction Models Using the Matched Molecular Pair Kernel
Source: Molecules. 2021 Aug 13;26(16):4916. doi: 10.3390/molecules26164916 (PMC8401777; doi:10.3390/molecules26164916)
Supplement: Supplementary file 1 [file molecules-26-04916-s001.zip › SupportingInformation.pdf]

Supporting Information

for

Interpretation of Ligand-based Activity Cliff Prediction Models using the Matched Molecular Pair Kernel

Shunsuke Tamura,[†] Swarit Jasial, [†,‡] Tomoyuki Miyao,[†,‡] and Kimito Funatsu[‡]

[†]Graduate School of Science and Technology, Nara Institute of Science and Technology, 8916-5 Takayama-cho, Ikoma, Nara, 630-0192, Japan

[‡]Data Science Center, Nara Institute of Science and Technology, 8916-5 Takayama-cho, Ikoma, Nara, 630-0192, Japan

*To whom correspondence should be addressed:

Tel: +81-3-5440-0396, Fax: +81-743-72-6037, E-mail: funatsu@dsc.naist.jp

Figure S1

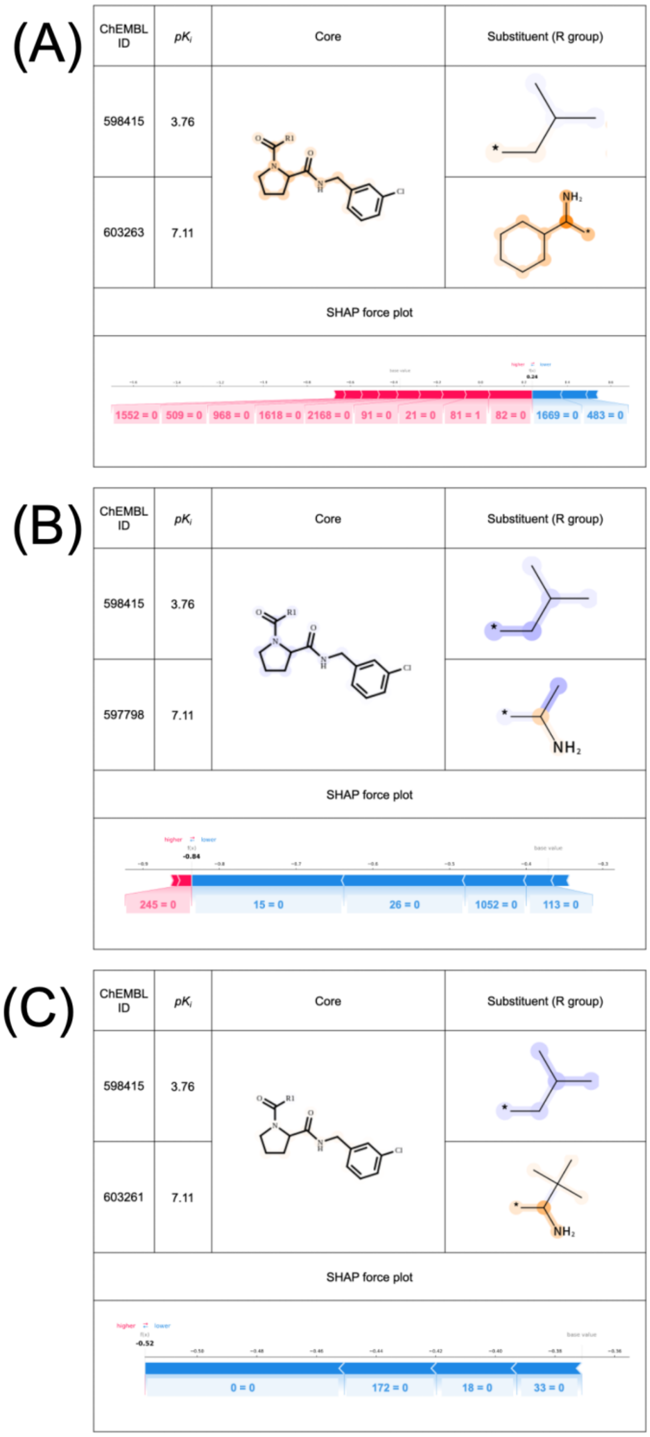

Figure S1. Comparison of feature contribution mapping and SHAP for exemplary thrombin MMPs predicted as TP, TN, FN. Exemplary (A) TP, (B) TN, and (C) FN MMPs of thrombin with feature contribution map and SHAP force plot for the MMPs is shown. MMPs are formed by (A) ChEMBL598415 and ChEMBL603263, (B) ChEMBL598415 and ChEMBL597798 and (C) ChEMBL598415 and ChEMBL603261.

Figure S2

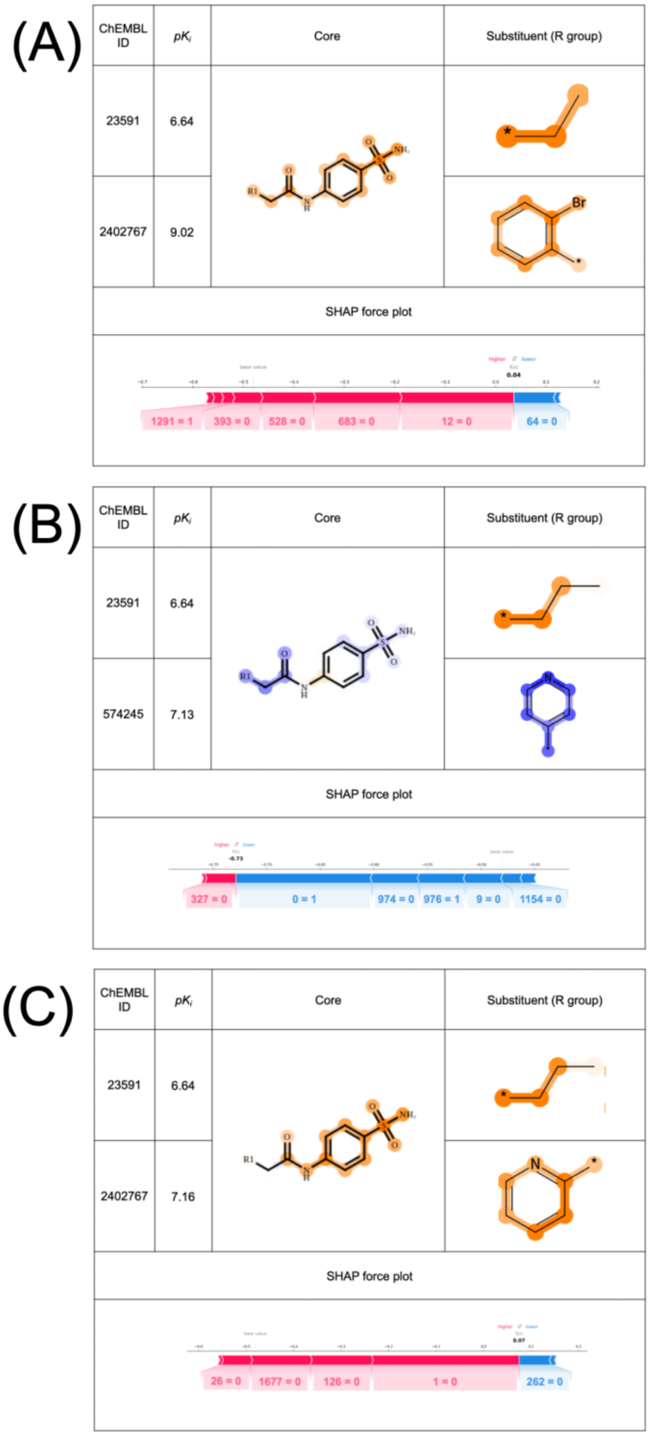

Figure S2. Comparison of feature contribution mapping and SHAP for exemplary ca2 MMPs predicted as TP, TN, FP. Exemplary (A) TP, (B) TN, and (C) FP MMPs of ca2 with feature contribution map and SHAP force plot for the MMPs is shown. MMPs are formed by (A) ChEMBL23591 and ChEMBL2402767, (B) ChEMBL23591 and ChEMBL574245 and (C) ChEMBL23591 and ChEMBL582965.
